# Regulation of the regenerative activity of dental pulp stem cells from exfoliated deciduous teeth (SHED) of children by TGF-β1 is associated with ALK5/Smad2, TAK1, p38 and MEK/ERK signaling

**DOI:** 10.18632/aging.103848

**Published:** 2020-11-04

**Authors:** Hsiao-Hua Chang, Il-Ly Chen, Yin-Lin Wang, Mei-Chi Chang, Yi-Ling Tsai, Wen-Chien Lan, Tong-Mei Wang, Sin-Yuet Yeung, Jiiang-Huei Jeng

**Affiliations:** 1 Department of Dentistry, National Taiwan University Hospital, and School of Dentistry, National Taiwan University Medical College, Taipei, Taiwan; 2 Chang Gung University of Science and Technology, Kwei-Shan, Taoyuan, Taiwan; 3 Department of Dentistry, Chang Gung Memorial Hospital, Taipei, Taiwan; 4 Department of Oral Hygiene Care, Ching Kuo Institute of Management and Health, Keelung, Taiwan; 5 School of Dentistry, College of Dental Medicine, Kaohsiung Medical University, Kaohsiung, Taiwan; 6 Department of Dentistry, Kaohsiung Medical University Hospital, Kaohsiung, Taiwan

**Keywords:** aging-related diseases, alkaline phosphatase, collagen, cyclooxygenase-2, differentiation

## Abstract

Transforming growth factor-β1 (TGF-β1) regulates wound healing/regeneration and aging processes. Dental pulp stem cells from human exfoliated deciduous teeth (SHED) are cell sources for treatment of age-related disorders. We studied the effect of TGF-β1 on SHED and related signaling. SHED were treated with TGF-β1 with/without pretreatment/co-incubation by SB431542, U0126, 5Z-7-oxozeaenol or SB203580. Sircol collagen assay, 3-(4,5-Dimethylthiazol-2-yl)-2,5- diphenyl tetrazolium bromide (MTT) assay, alkaline phosphatase (ALP) assay, RT-PCR, western blotting and PathScan phospho-ELISA were used to measure the effects. We found that SHED expressed ALK1, ALK3, ALK5, TGF-RII, betaglycan and endoglin mRNA. TGF-β1 stimulated p-Smad2, p-TAK1, p-ERK, p-p38 and cyclooxygenase-2 (COX-2) protein expression. It enhanced proliferation and collagen content of SHED that were attenuated by SB431542, 5Z-7-oxozeaenol and SB203580, but not U0126. TGF-β1 (0.5-1 ng/ml) stimulated ALP of SHED, whereas 5-10 ng/ml TGF-β1 suppressed ALP. SB431542 reversed the effects of TGF-β1. However, 5Z-7-oxozeaenol, SB203580 and U0126 only reversed the stimulatory effect of TGF-β1 on ALP. Four inhibitors attenuated TGF-β1-induced COX-2 expression. TGF-β1-stimulated TIMP-1 and N-cadherin was inhibited by SB431542 and 5Z-7-oxozeaenol. These results indicate that TGF-β1 affects SHED by differential regulation of ALK5/Smad2/3, TAK1, p38 and MEK/ERK. TGF-β1 and SHED could potentially be used for tissue engineering/regeneration and treatment of age-related diseases.

## INTRODUCTION

Human mesenchymal stem cells (MSCs) are multipotent progenitor cells that have the ability to self-renew and differentiate to different lineages of connective tissues, such as bone, cartilage, muscle and dentin [1]. Exfoliation of the deciduous teeth from children is a natural and necessary process for human growth and development. From the residual dental pulp tissue of shedding deciduous teeth, Miura et al. isolated multipotent stem cells and named them as stem cells from human exfoliated deciduous teeth (SHED) [2]. SHED show early mesenchymal stem cell markers such as STRO-1 and CD146, which are also found to be expressed in bone marrow stem cells (BMSCs) and human dental pulp stem cells (DPSCs). But SHED expressed little hematopoietic markers including CD14, CD34 and CD45 [3, 4]. SHED also demonstrate to express embryonic stem-cell markers, Oct-4 and Nanog, which are vital for the continuance of pluripotency in embryonic stem cells [5]. As compared with BMSCs and DPSCs, SHEDs show better performance in proliferation rate, telomerase activity and expression of genes participating in cell replication [2, 6, 7]. Transplantation of SHED *via* tail vein has been shown to prevent bone loss in ovariectomy-induced osteoporosis in experimental rats [8]. SHED is also applied in dermatology to improve dermal wound healing in response to photoaging [9]. In neural inductive environment, SHED may develop multiple long cytoplasmic processes and represent neurosphere-like cell clusters [10], with potential treatment for aging-related neurological diseases. In aged Parkinsonian rats, SHED is demonstrated to increase the population of dopaminergic neuron and alleviate behavior disorders [4, 11]. These findings suggest the neurogenic potential of SHED for treatment of age-related neurodegenerative diseases. SHED also exhibit significant immunomodulatory characteristics, which decrease the rejection confronting allograft transplantation, and even are feasible to treat immune diseases [7, 12]. According to the superior proliferation capacity, multipotent differentiation, immune modulatory property and noninvasive accessibility, SHED is considered an attractive stem cell source for tissue repair/engineering and regeneration for immune and other age-related diseases [13].

As potent stem cells derived from dental origin, the potential of SHED for dental tissue repair and regeneration is highly expected. When transplanted with hydroxyapatite/tricalcium phosphate (HA/TCP) into immunocompromised mice, SHED may differentiate to odontoblast-like cells, and form dentin-like tissue [2, 14]. SHED are demonstrated to generate dentin-pulp complex-like structure, which contained odontoblast-like cells lining on newly formed predentin, and angiogenic endothelial-like cells [15], potential for treatment of tooth and vascular diseases. Besides, SHED show evident osteogenic differentiation ability. They are found to produce bone tissue along the surface of HA/TCP thus decrease the calvarial defects of immunocompromised mice, and also enhanced proliferation of murine host bone-forming cells [2, 16, 17]. This bone induction effects may play beneficial roles in periodontal tissue regeneration and osteoporosis [18]. Recently, combination of cell sheets formed by SHED and treated dentin matrix has been demonstrated to induce periodontium-like tissue formation, including periodontal ligament fibers, blood vessels and bone [19]. Collectively, SHED have promising ability of odontogenesis, and may be accounted for cell source of regenerative endodontic and periodontal therapy.

Since SHED are capable of forming dentin-, pulp- and periodontium-like tissue, the underlying mechanism should be clarified. Repair of pulpo-dentin complex is controlled by processes including cell proliferation, chemotaxis, and differentiation into odontoblasts, leading to reparative dentin formation. Transforming growth factors-β1 (TGF-β1) is associated with aging-related disorders including Alzheimer's disease, muscle atrophy, and obesity [20]. Impairment of TGF-β signaling may contribute to aging diseases such as tissue degeneration, fibrosis, inflammation, osteoporosis and reduce the regeneration activity and metabolic dysfunction [20]. TGF-β1 may regulate both tooth development and the response to external irritation. In embryonic stage, TGF-β1 is expressed in dental epithelium and dental mesenchyme from bud stage to root formation, participating in the tooth organogenesis/morphogenesis [21]. During progression of dental caries, TGF-β1 residing in dentin matrix can be released by acidogenic bacteria, affects biological activities of dental pulp and regulates the reactionary and reparative dentin formation [22, 23]. Compared to healthy teeth, the odontoblasts and pulpal cells of carious teeth express increased level of TGF-β1 [24]. Furthermore, TGF-β1 stimulates dentinogenesis and enhances pulp repair when applied for direct pulp capping in rats and dogs [25, 26]. These results suggest TGF-β1 plays a role in response to pulpal injury and reparative process.

TGF-β1 regulates biological activities of cells by ligand-receptor binding and intracellular signaling cascades activation. TGF-β receptor type I (TGF-βRI) and type II (TGF-βRII) are the major parts of the signal transducer, and type III (TGF-βRIII) plays an auxiliary role by modulation of the ligand-binding specificity and affinity [27]. It firstly binds to TGF-βRII; TGF-βRI is then recruited, phosphorylated, and subsequently activates the downstream signal transduction [28]. There are seven TGF-βRI isoforms in humans, namely activin receptor-like kinase (ALK) 1-7, which show differential affinity with members of TGF-β superfamily [29]. The intracellular TGF-β signaling is conveyed mainly by Smad-dependent signaling pathways, which TGF-βRI transmits signals through phosphorylating receptor mediated R-Smads (Smad1, 2, 3, 5, 8), to regulate target genes expression [30]. Generally, TGF-β1 is considered to transmit signal primarily *via* ALK5 to activate Smad2/3 [28]. In previous studies TGF-β1 was found to down-regulates Runt-related transcription factor 2 (Runx-2) and alkaline phosphatase (ALP) in adult human dental pulp cells *via* ALK5/Smad2/3 signaling [31]. On the other side, TGF-β signaling can be mediated by Smad-independent pathways include various branches of mitogen-activated protein kinase (MAPK), TGF-β-activated kinase (TAK), Rho-like GTPase, and phosphatidylinositol-3-kinase (PI3K)/protein kinase-B (PKB, Akt) signaling pathways [32, 33]. It has been demonstrated that U0126, an inhibitor of Mitogen-activated protein kinase kinase-1/2 (MEK1/MEK2), suppresses the TGF-β1-induced cell proliferation and collagen formation in stem cells from apical papilla (SCAPs) [34]. The signaling pathways involved in the function and regenerative potential of SHED is still unclear and await exploration.

The proliferation of stem cells, deposition of various matrix proteins and cell differentiation to form mineralized structure are central to dental tissue regeneration. To learn more about activation of SHED during wound healing or tissue regeneration, we proposed analyses about the effects of TGF-β1 on SHED growth, collagen synthesis, osteo/odontoblastic differentiation, and associated cyclooxygenase-2 (COX-2), tissue inhibitor metalloproteinase-1 (TIMP-1), and N-cadherin expression. Activation of TGF-β receptors has been found to induce signal transduction pathways in adult dental pulp cells [35]. Both Smads kinase cascade and non-Smad signaling are shown to differentially mediate the effect of TGF-β1 in different kinds of cells [36, 37]. To further elucidate the signal transduction pathways for TGF-β1-induced changes in SHED is also the aim of this study. Clarifying these issues is beneficial for clinical application of SHED to regenerative dentistry, such as pulp/dentin repairing, root regeneration, and even total tooth generation. Moreover, SHED also can be potentially used for treatment of age-related diseases (osteoporosis, periodontal diseases, neurological diseases, Parkinsonism, cardiovascular diseases, and immune disorders) in the future.

## RESULTS

### Expression of stem cell markers in SHED

**Figure 1:**
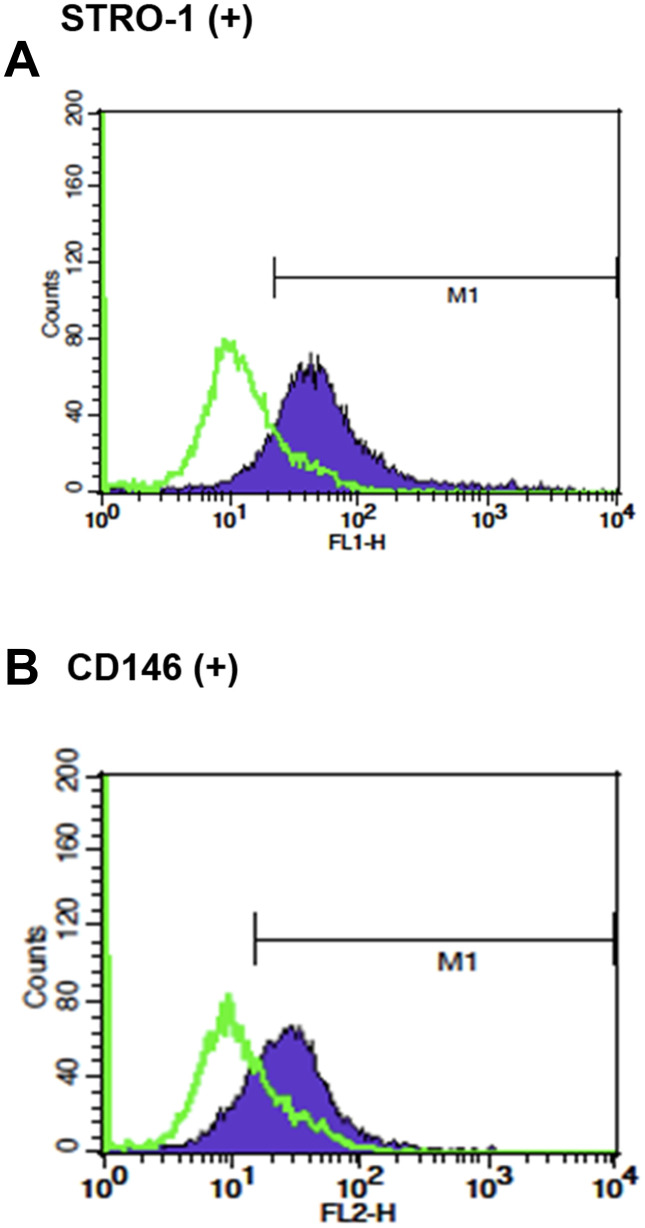
**The stem cell characteristics of SHED.** (**A**) Expression of STRO-1 and (**B**) expression of CD146 in cultured SHED. One representative picture of flow cytometric analysis data was shown.

Cultured SHED expressed early mesenchymal stem cell markers, STRO-1 (45.60 ± 26.83%) and CD146 (48.27 ± 24.72%), respectively as analyzed by flow cytometry analysis (Figure 1).

### Expression of TGF-β related receptors in SHED

**Figure 2:**
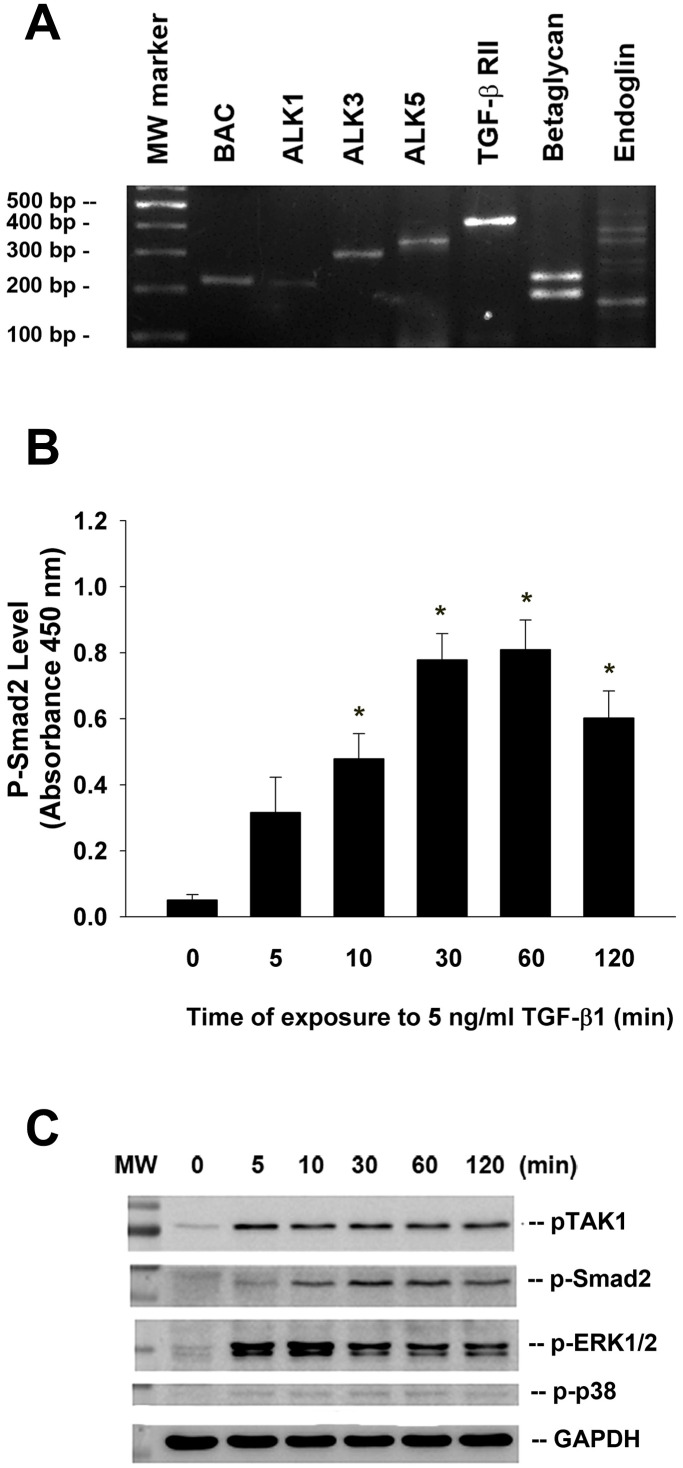
**Expression of various TGF-β related receptors in SHED and the effect of TGF-β1 on the Smad2, TAK1, ERK1/2 and p38 phosphorylation of SHED.** (**A**) SHED cells were cultured in DMEM with 10%FBS for 24 hours. Total RNA was isolated for RT-PCR analysis of TGF-β related receptors (ALK1, ALK3, ALK5, TGF-β1RII, betaglycan, endoglin) expression, (**B**) SHED were exposed to TGF-β1 for 0-120 min (as indicated on graphs). Cell lysates were prepared and proteins were used for analysis of p-Smad2 expression by PathScan phospho-ELISA (OD450, Mean ± SE). *Denotes statistically significant difference when compared with control. (**C**) SHED were exposed to TGF-β1 for 0-120 min. Cell lysates were prepared and proteins were used for western blotting analysis of p-Smad2, p-TAK1, p-ERK1/2, p-p38 and GAPDH (control) protein expression. One representative western blotting picture was shown.

Reverse transcription-polymerase chain reaction (RT-PCR) analysis of total RNA isolated from cultured SHED and found that TGF-β related receptors (ALK1, ALK3, ALK5, TGF-β RII, betaglycan, endoglin) expression were detectable in SHED cells (Figure 2A).

### Effect of TGF-β1 on Smad2, TAK1, ERK1/2, and p38 phosphorylation of SHED

Exposure of SHED to TGF-β1 rapidly stimulated Smad2 phosphorylation within 5-10 min of exposure, with a maximal stimulation at 30 to 60 min of incubation as analyzed by PathScan p-Smad2 enzyme-linked immunosorbent assay (ELISA) (Figure 2B). Accordingly, TGF-β1 also induced Smad2 phosphorylation as analyzed by western blotting. Similarly, TGF-β1 stimulated the phosphorylation and activation of TAK1, extracellular signal-regulated kinase 1/2 (ERK1/2) and p38 of SHED cells within 10 min of exposure, with a maximal stimulation of p-TAK1 and p-ERK1/2 at 5-10 min of exposure (Figure 2C).

### Effect of TGF-β1 on the proliferation of SHED

**Figure 3:**
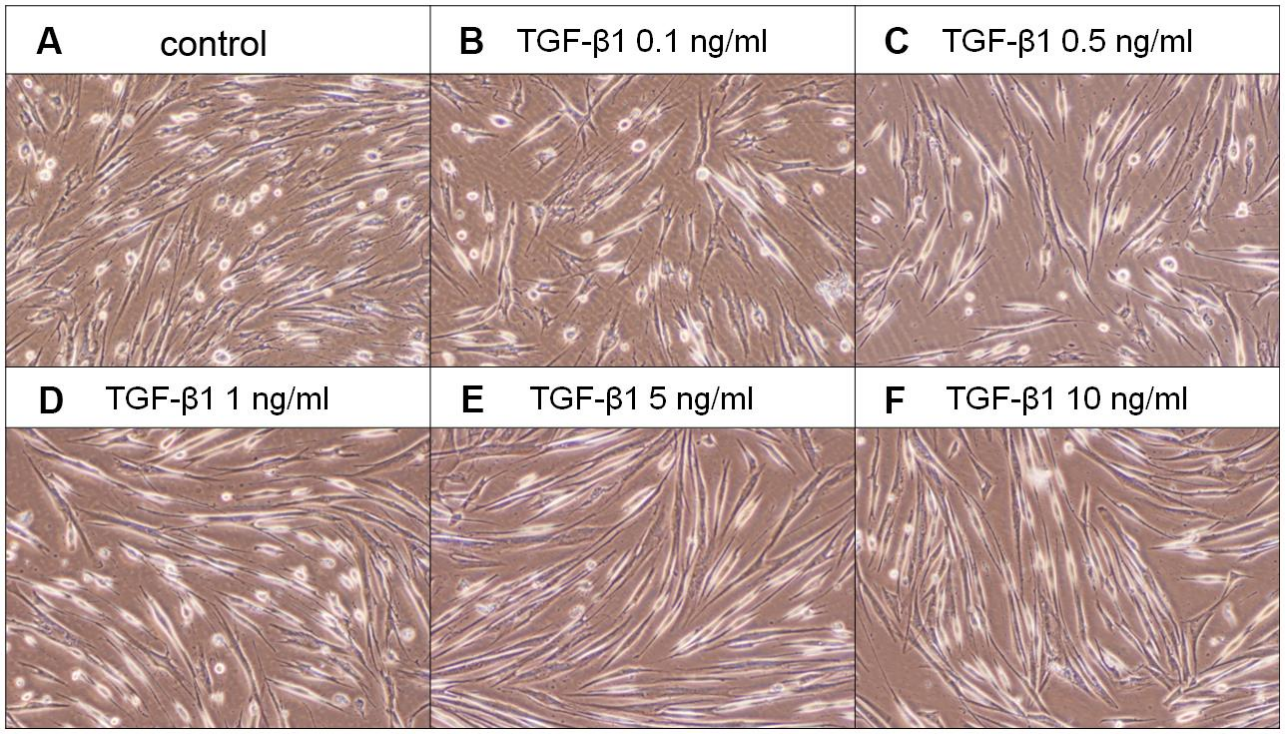
**Effect of TGF-β1 on the morphology of SHED.** (**A**) Control SHED, exposure to (**B**) 0.1 ng/ml TGF-β1, (**C**) 0.5 ng/ml TGF-β1, (**D**) 1 ng/ml TGF-β1, (**E**) 5 ng/ml TGF-β1 and (**F**) 10 ng/ml TGF-β1 for 5 days. One representative morphologic picture of cells was shown.

**Figure 4:**
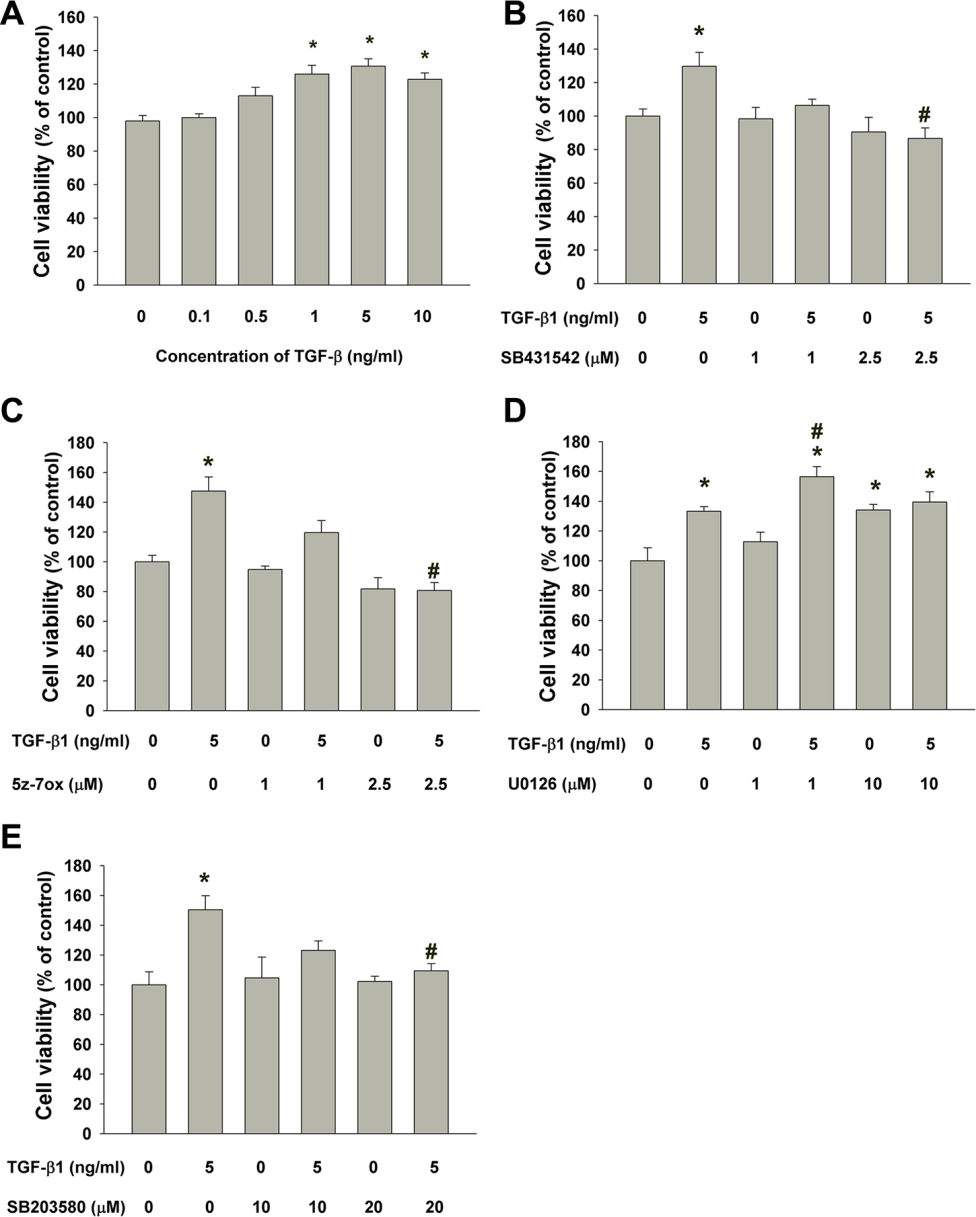
**Effect of TGF-β1 on the growth of SHED.** (**A**) Number of viable SHED after exposure to TGF-β1 for 5 days as estimated by MTT assay. Results were expressed as cell viability (% of control, Mean ± SE). (**B**) Effect of SB431542 on the TGF-β1-induced growth of SHED as analyzed by MTT assay. (**C**) Effect of 5z-7oxozeaenol on the TGF-β1-induced growth of SHED as analyzed by MTT assay. (**D**) Effect of U0126 on the TGF-β1-induced growth of SHED as analyzed by MTT assay. (**E**) Effect of SB203580 on the TGF-β1-induced growth of SHED as analyzed by MTT assay. *Denotes statistically significant difference when compared to control, #denotes statistically significant difference when compared with TGF-β1 (5 ng/ml)-treated group.

SHED cells are long and spindle shaped in appearance. No obvious morphologic change of SHED was noted after exposure to TGF-β1 (0.1-0.5 ng/ml). SHED became thinner and longer in appearance when exposed to 1-10 ng/ml of TGF-β1 for 5 days (Figure 3). TGF-β1 stimulated the growth of SHED at concentrations of 1-10 ng/ml (Figure 4A). Pretreatment and co-incubation with SB431542, 5Z-7-oxozeaenol and SB203580 markedly attenuated the TGF-β1-stimulated growth of SHED (Figure 4B, 4C, 4E), whereas U0126 showed little effect and even enhanced this event (Figure 4D).

### Effects of TGF-β1 on COX-2 protein expression of cultured SHED

**Figure 5:**
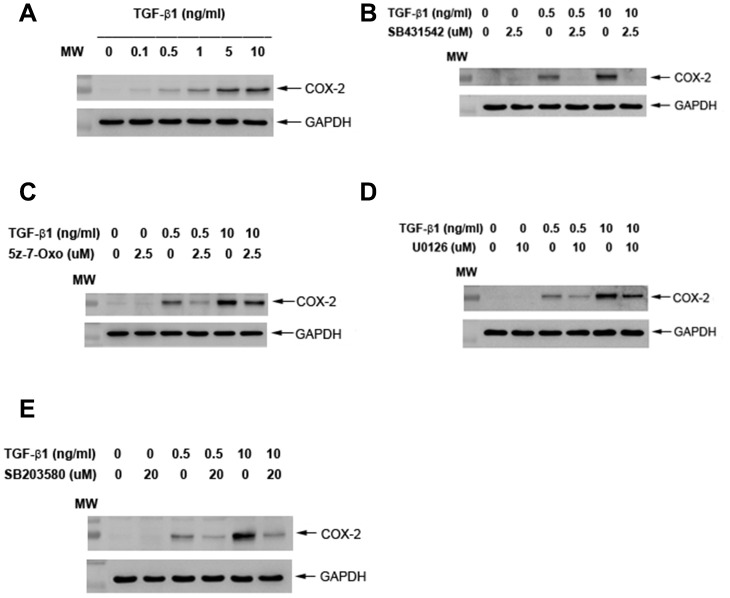
**Effect of TGF-β1 on COX-2 protein expression of cultured SHED.** (**A**) COX-2 protein expression of SHED after exposure to TGF-β1 for 24 hours. (**B**) Effect of SB431542 on the TGF-β1-induced COX-2 expression of SHED. (**C**) Effect of 5z-7oxozeaenol on the TGF-β1-induced COX-2 expression of SHED. (**D**) Effect of U0126 on the TGF-β1-induced COX-2 expression of SHED. (**E**) Effect of SB203580 on the TGF-β1-induced COX-2 expression of SHED. One representative western blot picture was shown.

Exposure to TGF-β1 induced the COX-2 protein expression of SHED at concentrations of 0.5 ng/ml or higher (1-10 ng/ml) (Figure 5A). Pretreatment and co-incubation by SB431542 (2.5 μM), 5z-7-oxozeaenol (2.5 μM), U0126 (10 μM) and SB203580 (20 μM) all markedly attenuated the TGF-β1 (0.5 and 10 ng/ml)-induced COX-2 protein expression of SHED cells (Figure 5B–5E).

### Effects of TGF-β1 on collagen content of cultured SHED

**Figure 6:**
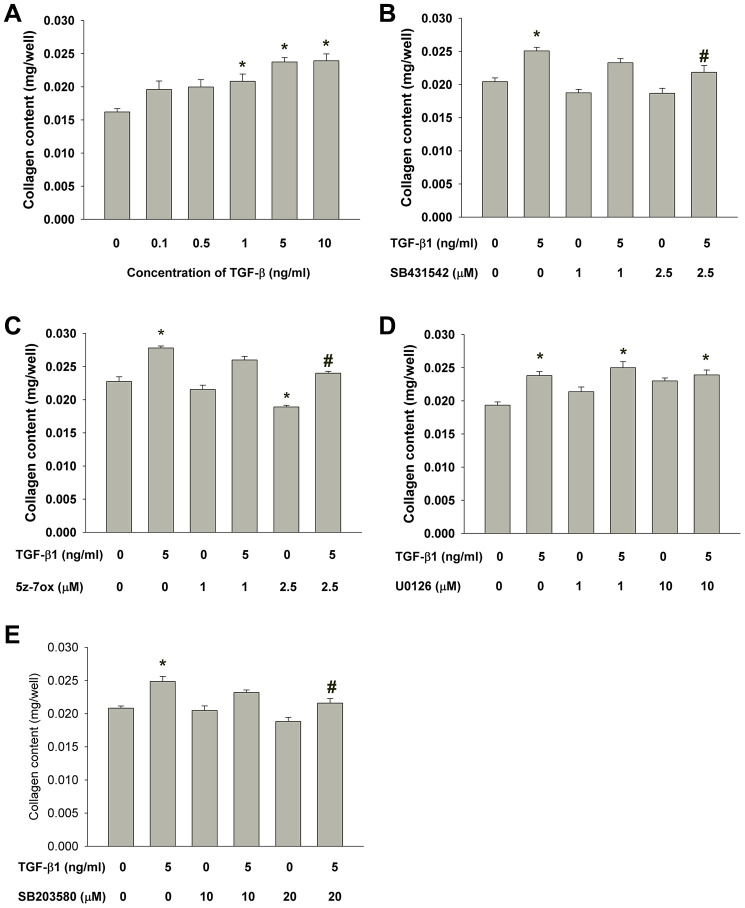
**Effect of TGF-β1 on the collagen content of cultured SHED.** (**A**) Collagen content of SHED after exposure to TGF-β1 for 5 days as measured by Sircol collagen assay. Results were expressed Mean ± SE. (**B**) Effect of SB431542 on the TGF-β1-induced increase in collagen content of SHED. (**C**) Effect of 5z-7oxozeaenol on the TGF-β1-induced increase in collagen content of SHED. (**D**) Effect of U0126 on the TGF-β1-induced increase in collagen content of SHED. (**E**) Effect of SB203580 on the TGF-β1-induced increase in collagen content of SHED. *Denotes statistically significant difference when compared to control, #denotes statistically significant difference when compared with TGF-β1 (5 ng/ml)-treated group.

Exposure to TGF-β1 stimulated the collagen content of cultured SHED. As shown in Figure 6A, the collagen content in monolayer culture of SHED evidently increased after exposure to higher concentrations of TGF-β1 (1-10 ng/ml). The TGF-β1-induced increase in collagen content of SHED could be suppressed by SB431542, 5Z-7-oxozeaenol and SB203580 but not U0126 (Figure 6B–6E).

### Effect of TGF-β1 on Alkaline phosphatase (ALP) activity of SHED

### Alkaline phosphatase (ALP) staining

**Figure 7:**
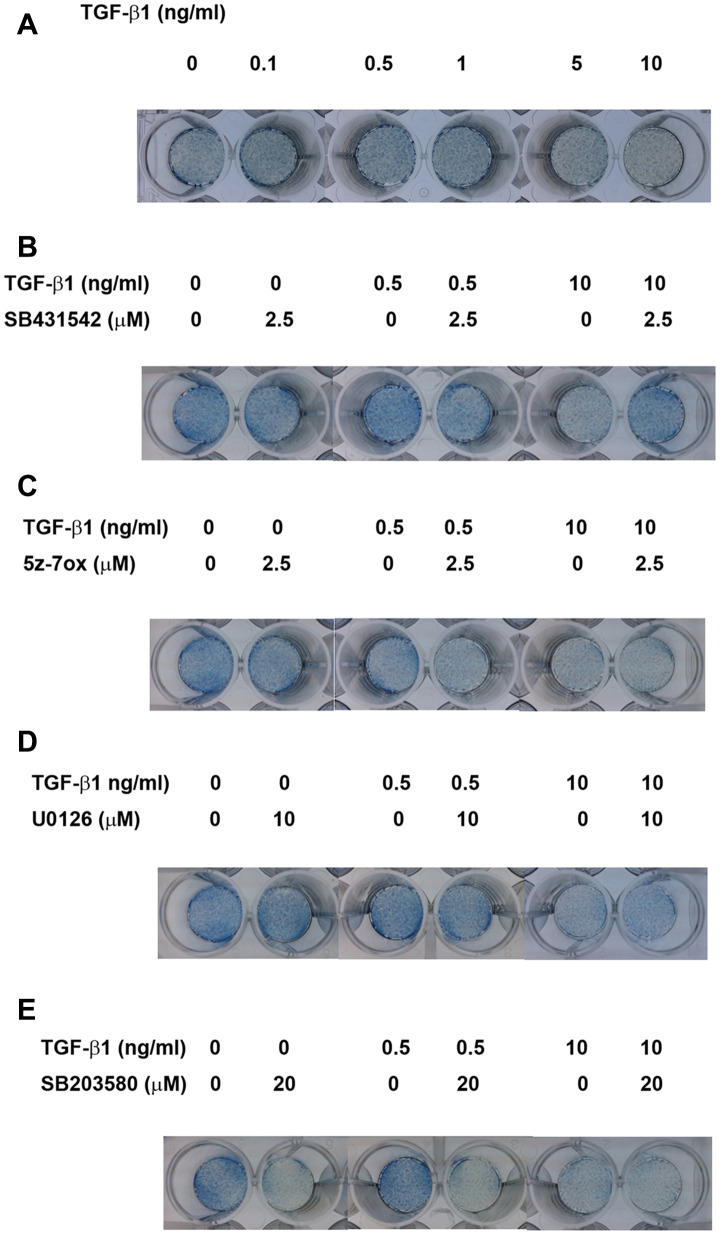
**Effect of TGF-β1 on the ALP activities of cultured SHED as analyzed by ALP staining.** (**A**) ALP staining of SHED after exposure to TGF-β1 for 5 days. (**B**) Effect of SB431542 on the TGF-β1-induced increase or decrease in ALP activity of SHED. (**C**) Effect of 5z-7oxozeaenol on the TGF-β1-induced increase or decrease in ALP activity of SHED. (**D**) Effect of U0126 on the TGF-β1-induced increase or decrease in ALP activity of SHED. (**E**) Effect of SB203580 on the TGF-β1-induced increase or decrease in ALP activity of SHED. One representative ALP staining result was shown.

As shown in Figure 7A, at concentrations of 0.5-1 ng/ml, TGF-β1 up-regulated the ALP staining of SHED after 5 days of exposure. In addition, TGF-β1 suppressed the ALP staining of SHED at concentrations of 5 and 10 ng/ml. SB431542 prevented the TGF-β1 (0.5 ng/ml)-induced increase in ALP activity and also reversed the TGF-β1 induced decline in ALP activity (Figure 7B). On the other hand, 5Z-7-oxozeaenol, U0126 and SB203580 inhibited the TGF-β1 (0.5 ng/ml)-induced increase in ALP activity, but showed little preventive effect on TGF-β1 (10 ng/ml)-induced decline in ALP activity (Figure 7C–7E).

### ALP enzyme activity assay

**Figure 8:**
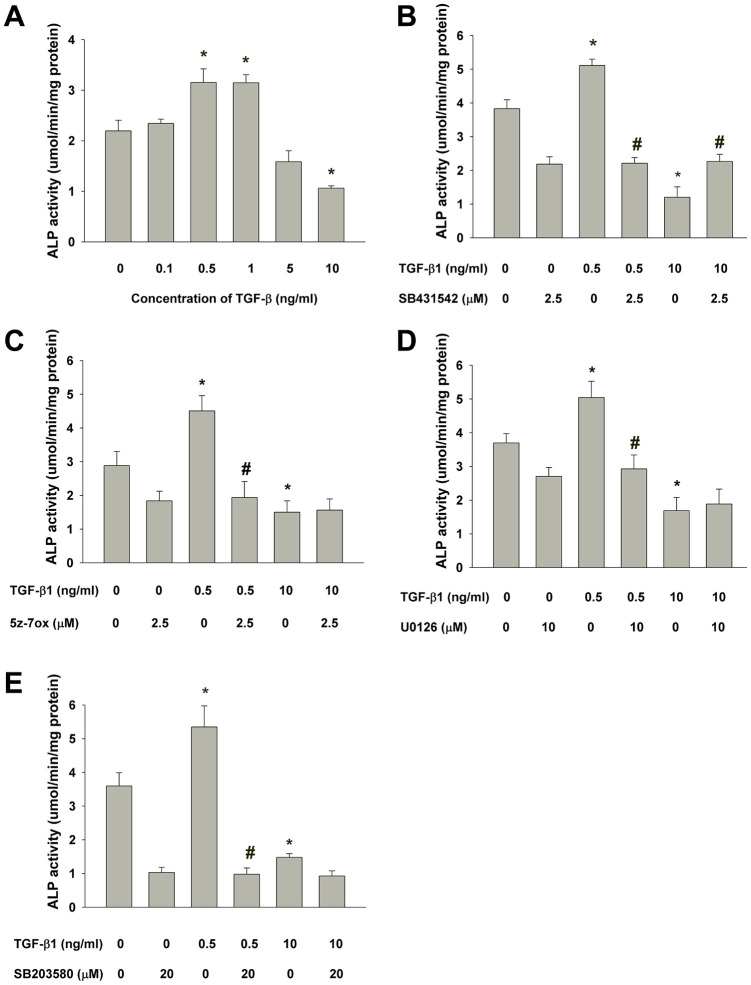
**Effect of TGF-β1 on the ALP activities of cultured SHED as analyzed by ALP enzyme activity assay.** (**A**) Quantitative ALP enzyme activity assay of SHED with/without exposure to TGF-β1 for 5 days. (**B**) Effect of SB431542 on the TGF-β1-induced increase or decrease in ALP activity of SHED. (**C**) Effect of 5z-7oxozeaenol on the TGF-β1-induced increase or decrease in ALP activity of SHED. (**D**) Effect of U0126 on the TGF-β1-induced increase or decrease in ALP activity of SHED, (**E**) Effect of SB203580 on the TGF-β1-induced increase or decrease in ALP activity of SHED. *Denotes statistically significant difference when compared with respective control group. #Denotes statistically significant difference when compared with respective TGF-β1-treated group.

Quantitatively, ALP enzyme activity was determined and showed that TGF-β1 (0.5-1 ng/ml) also stimulated ALP activity of SHED after 5 days of exposure, but suppressed the ALP activity at a higher concentration (10 ng/ml) (Figure 8A). To further clarify by which pathway that TGF-β1 regulates the ALP activity of SHED, SB431542 was intriguingly found to prevent both the TGF-β1 (0.5 ng/ml)-induced increase in ALP activity and also reversed the TGF-β1-induced decline in ALP activity (Figure 8B). Moreover, 5z-7oxozeaenol, U0126 and SB203580 was shown to inhibit the TGF-β1 (0.5 ng/ml)-induced increase in ALP activity, but exhibit little preventive effect on the TGF-β1 (10 ng/ml)-induced decline in ALP activity (Figure 8C–8E).

### Effects of TGF-β1 on TIMP-1 and N-cadherin protein expression of cultured SHED

**Figure 9:**
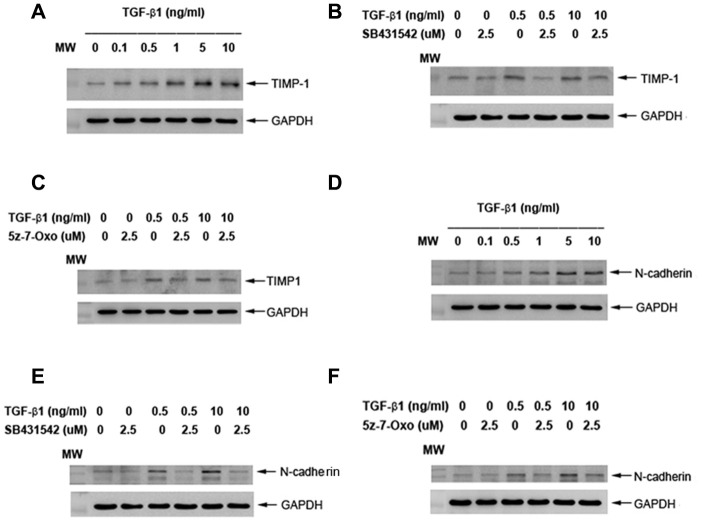
**Effect of TGF-β1 on TIMP-1 and N-cadherin protein expression of cultured SHED.** (**A**) TIMP-1 protein expression of SHED after exposure to TGF-β1 for 24 hours. (**B**) Effect of SB431542 on the TGF-β1-induced TIMP-1 expression of SHED. (**C**) Effect of 5z-7oxozeaenol on the TGF-β1-induced TIMP-1 expression of SHED. (**D**) N-cadherin protein expression of SHED after exposure to TGF-β1 for 24 hours. (**E**) Effect of SB431542 on the TGF-β1-induced N-cadherin expression of SHED. (**F**) Effect of 5z-7oxozeaenol on the TGF-β1-induced N-cadherin expression of SHED. One representative western blot picture was shown.

To know more about TGF-β1 on collagen turnover and differentiation, we found that exposure to TGF-β1 stimulated the TIMP-1 protein expression of SHED at concentrations of 0.1-10 ng/ml (Figure 9A). Pretreatment and co-incubation by SB431542 (2.5 μM), 5z-7-oxozeaenol (2.5 μM), obviously suppressed the TGF-β1 (0.5 and 10 ng/ml)-induced TIMP-1 protein expression of SHED (Figure 9B, 9C). Accordingly, TGF-β1 stimulated the N-cadherin (an odontoblast differentiation marker) protein expression of SHED at concentrations of 0.5-10 ng/ml (Figure 9D). Pretreatment and co-incubation by SB431542 (2.5 μM), 5z-7-oxozeaenol (2.5 μM), also evidently attenuated the TGF-β1-induced N-cadherin protein expression of SHED (Figure 9E, 9F).

## DISCUSSION

Dental pulp is basically a loose connective tissue confined in the chamber and root canal(s) within a tooth. In 2000, mesenchymal stem cells were firstly separated from pulp of human permanent teeth and termed as dental pulp stem cells (DPSCs) [38]. Pulp tissues in primary teeth show similar histological features with permanent teeth [39]. From an analogous pattern and much more non-invasive access, SHED from exfoliated teeth of children were thus discovered and demonstrated with better clonogenicity and proliferation rate [2]. Due to the primitive origin, SHED also show more stemness characters and differentiation ability to endothelial cells, odontoblasts and osteoblasts- like cells for repair and regeneration of aging-related diseases. In the present study, we cultured SHED and analyzed the expression of STRO-1 and CD146, which have been pervasively used as early mesenchymal stem cell markers. The percentages of cultured SHED expressing STRO-1 and CD146 were 45.6 % and 48.3% respectively, which were higher than DPSCs and SCAPs reported [34, 40]. Shi et al. have found most colony-forming cells in dental pulp represent STRO-1 and CD146 [41]. Our results suggested the primitivity and clonogenicity of cultured SHED.

For gaining insights into the mechanism of TGF-β1 effect on SHED, the signaling operation need to be explored. Here we found SHED express many TGF-β1 signaling related receptors: TGF-βRIs (ALK1, ALK3, ALK5), TGF-βRII, betaglycan and endoglin were all clearly detected. Recently these receptors were also found to be expressed in SCAPs in a non-stimulated condition, and TGF-β1 activated Smad2 phosphorylation in a quick and dose-dependent mode [34]. Similarly, we observed that the expression of p-Smad2 was observed as early as 5 min after the treatment of TGF-β1 (5 ng/ml) and increased to the peak at 60 min, and extend for more than 120 min. These results suggested these receptors and the canonical Smad-dependent pathway mediated TGF-β1-induced signaling in SHED. Besides Smad2, TGF-β1 also rapidly induced expression of phosphorylated TAK1, ERK1/2 and p38, as revealed by western blot. How these Smad-independent signaling factors involve in TGF-β1-induced effect on SHED deserves further exploration. In the present study, we observed cell viability was up-regulated by treatment of 0.5-10 ng/ml TGF-β1, in a serum-free condition. Prior studies have reported TGF-β1 has potent anti-proliferative effect on various cell types, such as epithelial, lymphoid and myeloid cells [42, 43]. However, TGF-β1 is also found to stimulate the growth of cells from mesenchymal origin, such as smooth muscle cells, immortalized fibroblasts and osteoblasts [44, 45]. Even for mesenchymal cells in dental tissues, which are all derived from neural crest mesenchyme, contradicted effect of TGF-β1 on cell viability are often noticed. In human dental pulp cells, TGF-β1 mildly decreases cell viability as cultured for 5 days in Dubelcco’s modified Eagle’s medium (DMEM) containing 10% fetal bovine serum (FBS) [37, 46]. On the contrary, stimulatory effect has also been reported: Shirakawa et al. showed 0.1-5 ng/ml TGF-β1 increasd dental pulp cell proliferation as measured by DNA content after 18-day culture, as TGF-β1 was added at day 10 [47]. In addition, the proliferation of isolated STRO-1 positive DPSCs is enhanced by TGF-β1 and reaches its peak at the concentration of 5 ng/ml [48]. In a serum-free condition, TGF-β1 (0.1-10 ng/ml) enhances SCAPs vitality in a dose-dependent manner [34]. Whilst in mineralizing differentiation medium, TGF-β1 seems to inhibit the growth of cultured SCAPs [49]. These divergent results caused by TGF-β1 may be related to cell origin, concentrations of TGF-β1, timing of administration, stem cell properties, the cell culture conditions, and the differential expression of cellular TGF-β signaling molecules. Here we found TGF-β1-induced mitogenic effect on SHED could be reversed by SB431542 (2.5μM), 5Z-7-Oxozeaenol (2.5 μM), and SB203580 (20 μM); nevertheless, it was not suppressed by U0126 (1, 10 μM). Previous studies demonstrate that TAK1 is required for TGF-β1 to induce the activation of p38 MAPK and c-jun N-terminal kinase (JNK) [50]. The findings suggest the stimulating effect of TGF-β1 on cell viability of SHED and can be potentially used for tissue repair and wound healing. These events were through ALK5-Smad2/3, TAK1 and p38 MAPK signaling, but not MEK/ERK pathway.

TGF-β, COX and prostanoids are important in early cutaneous wound healing response to injury as transient inflammatory reactions, followed by repair and healing [51, 52]. In addition to early/transient inflammatory effect, COX-2 and prostaglandin E_2_ (PGE_2_) are also shown to mediate the TGF-β-induced stemness of cancer cells and replenishment of endogenous cardiac stem cells after infartion injury to heart [53, 54]. COX-2, bone morphogenetic protein-2 (BMP-2) amd TGF-β have been shown to promote osteoblast differentiation of mesenchymal stem cells [55]. PGE_2_ also stimulates BMP-2 expression of mesenchymal stem cells *via* activation of EP4 receptors [56]. Moreover, PGE_2_ generated by murine stem cells are shown to suppress T cell proliferation and cellular immunity for treatment of colitis [57]. The roles of COX-2 in responsible for TGF-β1-induced events in SHED is not clear and awaits further investigation. TGF-β1 is shown to stimulate COX-2 *via* Smad-dependent pathway in granulosa cells [58]. We further found that four inhibitors are able to attenuate TGF-β1-induced COX-2 expression, suggesting the involvement of ALK5/Smad2, TAK1, MEK/ERK and p38 in this event.

The response of pulp repair and tissue regeneration, like wound healing of soft tissue, is related to cell chemotaxis, proliferation and extracellular matrix production. Collagen is well-known as the main protein component of extracellular matrix, and widely distributed in pulp tissue and dentin. Furthermore, increased synthesis of type I collagen is considered to be crucial for the differentiation of odontoblasts [59, 60]. Sircol collagen assay is pervasively used to measure collagen content by analysis of acid-soluble fibrillar collagens. By the assay we observed that the amount of collagen content of SHED was increased by 1-10 ng/ml TGF-β1, in a dose-related pattern. The enhancement of collagen production by TGF-β1 has been demonstrated in dental pulp cells and SCAPs recently, and the effect is mediated by both Smad2/3 and MEK/ERK signaling [34, 61]. However, we did not find the role of MEK/ERK signaling in our experiment, because the administration of U0126 (MEK/ERK inhibitor) did not impact the TGF-β1-induced collagen production in SHED. Instead, inhibition of Smad2, TAK1 and p38 significantly attenuated the increase of collagen content. These results suggest that matrix formation of SHED, like cell proliferation, can be enhanced by TGF-β1 which are mediated by ALK5-Smad2/3, TAK1 and p38 MAPK pathways. These findings imply TGF-β1 may be beneficial for SHED potential in pulp repair and regeneration, and the effect can be modulated by Smad2/3, TAK1, p38 MAPK signaling molecules. The higher expression of collagen and TIMP-1 in SHED has been reported [62]. In this study, increase of collagen content by TGF-β1 can be partly explained by its stimulation of TIMP-1 that may inhibit the matrix metalloproteinases (MMPs) activity for collagen degradation [63]. In addition, the stimulation of TIMP-1 expression by TGF-β1 in SHED is found to be associated with ALK5/Smad and TAK1 signaling.

ALP functions to regulate phosphate transport during calcified tissues (bone, dentin, cementum etc.) formation. Expression of ALP is regarded as an early marker of extracellular matrix deposition in odontogenesis [64]. Moreover, ALP activity represents highest level in the subodontoblastic layer, indicating ALP is essential for differentiation of odontoblasts [59]. Therefore, we analyzed the expression of ALP activity to assess the effect of TGF-β1 on odontogenic/osteogenic differentiation potential of SHED. In the present study, SHED were cultured in medium without mineralization-induction agents, such as ascorbic acid, dexamethasone or beta-glycerophosphate which may modulate the expression of ALP, for observing the influence of TGF-β1 solitarily. Interestingly, we found TGF-β1 may induce opposite effects while administered in different concentrations. After treatment of 0.5-1 ng/ml TGF-β1, ALP activity of cultured SHED increased obviously; however, 5-10 ng/ml TGF-β1 significantly decreased ALP expression, as revealed by ALP stain and quantitative assay. It has been reported that TGF-β1 inhibited ALP activity and gene expression in human dental pulp cells as treated at concentration 5-10 ng/ml, but no stimulatory effect was observed at 1 ng/ml [31]. Nevertheless, Shirakawa et al. has reported that 0.1 ng/ml TGF-β1 increased ALP activity in one strain of their cultured pulp cells, but not in other 3 strains, and 1-5 ng/ml TGF-β1 reduced ALP activity in all strains to a very low level [47]. These inconsistent results may suggest variance between human dental pulp cells, and infer different sensitivity and/or mechanism of TGF-β1-inducing effect on ALP activity from SHED. On the other hand, very comparable results was observed in SCAPs, of which ALP activity was enhanced by 0.1-1 ng/ml TGF-β1 and down-regulated at 5-10 ng/ml [34]. This similarity may result from parallel stem cell properties in SHED and SCAPs. Regard to signaling, we noticed that SB431542 could reverse the effect on ALP activity by TGF-β1 at both low and high concentrations, while U0126, 5-Z-7-Oxozeaenol and SB203580 only suppress the ALP-enhancing effect of TGF-β1. These findings suggested that Smad2/3 played a major role in the regulation of ALP activity by TGF-β1 in SHED, and MEK/ERK, TAK1 and p38 also participated in the signaling in specific concentrations, thus modulated the ALP expression. In dental pulp cells, SB431542 could overturn the TGF-β1-induced declination of ALP activity, but U0126 could not [31]; SB203580 could inhibit ALP activity induced by TGF-β1, and was capable to inhibit ALP activity in the absence of TGF-β1 [65]. As in SCAPs, the up- and down-regulated effects are reversed by SB431542 but U0126 can only decrease the TGF-β1-induced ALP enhancing effect, which agrees with our results [34]. In the present study, we also noticed that hindrance of these signaling pathways seemed to somehow interfere the basic ALP activity of SHED, especially the blockage to p38, which drastically reduced the ALP expression. Similar result has been demonstrated in dental pulp cells and osteoblast previously [65, 66]. Thus we conjectured, p38 MAPK, which can be activated by environmental stress and various cytokines, may be critical to SHED in the expression of ALP expression. Intriguingly TGF-β1 was further shown to induce N-cadherin expression of SHED *via* ALK5/Smad2 and TAK1 signaling. N-cadherin, as a calcium-dependent cell adhesion molecule associated with cell-cell and cell-matrix interaction [67]. It also regulates a number of biological processes such as cell recognition, intercellular communication, cell fate, cell polarity, boundary formation, and morphogenesis [68]. N-cadherin plays important roles in mineralization process of odontoblasts, ameloblasts, osteoblasts [69, 70]. No prior report has addressed its expression and role in SHED. In developing teeth, N-cadherin expressed is higher in cap and bell stages. Its expression is found mainly in differentiated epithelial cells, odontoblasts to product mineralized matrix [68]. These results suggest the possible role of TGF-β1 in induction of SHED differentiation and mineralization for treatment of age-related diseases such as osteoporosis, periodontal regeneration, pulp necrosis etc.

## CONCLUSIONS

**Figure 10:**
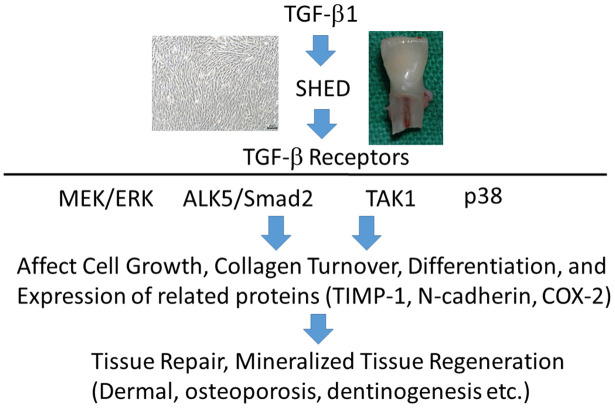
**TGF-β1 binds to TGF-β receptors of SHED to stimulate downstream signaling pathways such as ALK5/Smad2, TAK1, p38 and MEK/ERK to regulate growth, differentiation etc, can be potentially applied for treatment of aging-related diseases including dermal aging, osteoporosis, and pulp necrosis etc.**

SHED expressed TGF-β signaling-related receptors, including ALK1, ALK3, ALK5, TGF-β RII, betaglycan and endoglin. TGF-β1 stimulates ALK5/Smad2, TAK1, MEK/ERK, and p38 signaling pathways (Figure 10). It also significantly enhanced proliferation and collagen production of SHED, and both effects were attenuated by SB431542, 5Z-7-oxozeaenol and SB203580, but not by U0126. TGF-β1 (0.5-1 ng/ml) stimulated ALP activity of SHED, whereas 5-10 ng/ml TGF-β1 suppressed ALP expression. SB431542 could reverse the effects of TGF-β1 at both low and high concentrations. However, 5Z-7-oxozeaenol, SB203580 and U0126 only reduced the stimulatory effect of TGF-β1 (0.5-1 ng/ml) on ALP. All four signaling inhibitors attenuated the TGF-β1-induced COX-2 expression. These results indicate that TGF-β1 may regulate SHED behaviors such as proliferation, collagen turnover and differentiation. These events are through binding to TGF-β receptors and differentially regulated by ALK5/Smad2/3, TAK1, p38 and MEK/ERK signaling pathways. TGF-β1 and SHED can be potentially used for repair and regeneration for aging related diseases such as pulp necrosis, periodontitis, dermal aging, osteoporosis, and diseases of tissues/organs.

## MATERIALS AND METHODS

### Materials

3-(4,5-dimethylthiazol-2-yl)-2,5 diphenyl tetrazolium bromide (MTT), alkaline phosphatase (ALP) staining reagents and activity assay kits, and dimethylsulfoxide (DMSO) were purchased from Sigma/Aldrich (Sigma Chemical Company, St. Louis, MO, USA). Recombinant TGF-β1 was acquired from PeproTech Inc. (NJ, USA). Dulbecco’s modified Eagle’s medium (DMEM), fetal bovine serum (FBS), penicillin/streptomycin were obtained from Life Technologies (Thermafisher Scientific, Waltham, MA, USA). Sircol^TM^ collagen assay kit was from Biocolor Ltd. (Newtownabbey, Northern Ireland). RNA isolation kits were purchased from Macherey-Nagel (Macherey-Nagel Inc, Easton, PA, USA). The SuperScript TM III First-Strand DNA synthersis system, primary antibody mouse anti-human STRO-1 antibody, secondary antibody goat antimouse immunoglobulin M–FITC antibody were from Invitrogen (Invitogen Corporation, Carlsbad, CA, USA), and CD146 antibody was from BD (Biolegend). PathScan phospho-Smad2 enzyme-linked immunosorbant assay kits and p-Smad2 antibody were from Cell Signaling Technology (Danvers, MA, USA). SB431542, 5z-7-oxozeaenol, U0126, and SB203580 were purchased from Tocris Bioscience (Minneapolis, MN, USA). Antibodies for p-p38, p-ERK1/2, COX-2, N-cadherin and GAPDH were from Santa Cruz (Dallas, Texas, USA), whereas p-TAK1 and TIMP-1 antibodies were from GeneTex International Corporation (Hsinchu City, Taiwan).

### Culture of human SHED

Human primary teeth were extracted during natural shedding by the approval of Ethics Committee, National Taiwan University Hospital and written informed consent from the patients or next of kin on behalf of all minors enrolled in this study. The dental pulp tissues of primary teeth were obtained by a periodontal curette, minced into small pieces (about 1 x 1 x 1 mm^3^), and placed into 10-cm culture dishes. They were cultured by tissue explant technique in DMEM containing 10% FBS, 1% penicillin/streptomycin at 37°C in a humidified atmosphere of 5% CO_2_ and 95% air. When the outgrowth of SHED from tissue explant was near confluence in culture dishes, they were passaged at a ratio of 1:3. The SHED in passage numbers from 3 to 8 were used for this study. Three strains of SHED were cultured and used in this study.

### Characterization of surface markers in SHED

The expression of surface markers, STRO-1 and CD146 in SHED was analyzed by flow cytometric analysis at passages of 3-7 similar to previously [34]. Briefly, the SHED were incubated with mouse antihuman STRO-1 antibody (1:10) for 30 minutes, and then incubated for 30 minutes with a secondary antibody goat anti-mouse immunoglobulin M–FITC antibody (1:50). Cells were also incubated in R-PE conjugated monoclonal anti-human antibodies CD146 (1:50). Cells were washed twice with 2% FBS/Phosphate-buffered saline (PBS) and immediately subjected to flow cytometry analysis for the expression of STRO-1 and CD146 in SHED.

### Expression of various TGF-β receptors in SHED

The presence of TGF-β receptor family members including ALK1, ALK5, ALK3 (BMPRIA), TGF-βRII, betaglycan (TGF-βRIII) and endoglin were evaluated in SHED. Briefly, SHED cells were incubated in 10 cm culture dish with DMEM and 10% FBS for 24 hours. Total RNA was extracted and used for reverse-transcription polymerase chain reaction (RT-PCR). Specific primers used in this study were ALK1: 5’-ACAACATCCTAGGCTTCATCGCCT-3’ and 5’-TGGTTTGCCCTGTGTACCGAAGAT-3’ (212 bp), ALK5: 5’-GGGGCGACGGCGTTACAGTGTTTCTGCCAC-3’ and 5’-TGAGATGCAGACGAAGCACACTGGTCCAGC-3’ (333 bp) [71], ALK3 (BMPRIA): 5’-TAAAGGTGACAGTACACAGGAAACA-3’ and 5’-TCTAT GATGGCAAAGCAATGTCC-3’ (298 bp), TGF-β-RII: 5’-CGCGTTAACCGGCAGCAGAAG-3’ and 5’-GCGGTGATCAGCCAGTATTGTTT-3’ (410 bp) [72], betaglycan (TGF-βRIII): 5’-TGTGTGCCTCCTGACGAAGC-3’ and 5’-AGGCTGCAAACGCAATGCCC-3’ (217 bp) [73]. Primers for endoglin were GAATTCTGGTACATCTACTCGC and GGCTATGCCATGCTGCTGGTGG (150 bp and 285 bp) [74]. The primer sequence of beta-actin (BAC, 218 bp) is described as before [75] The PCR procedures were denaturing at 94°C for 30 seconds, annealing at 55°C for 30 seconds, and elongation at 72°C for 30 seconds for 35 cycles. The PCR generated products were subjected to 1.8% agarose gel electrophoresis and visualized after ethidium bromide staining.

### Effect of TGF-β1 on COX-2, TIMP-1, N-cadherin and phosphorylated ERK1/2, TAK1, p38 and Smad2 protein expression of SHED

Western blotting was used to measure the status of COX-2 and ERK1/2, TAK1, p38 and Smad2 phosphorylation. In short, 1.5 x 10^6^ SHED/10-cm dishes were exposed to TGF-β1 for 5, 10, 30, 60 and 120 min. Cell lysates were prepared as described previously [34, 75, 76]. The same amount of proteins was loaded for 12% polyacrylamide gel electrophoresis and transferred to Polyvinylidene Difluoride (PVDF) membranes (Millipore Sigma, Billerica, MA, USA). The membranes were immersed in a blocking reagent (20 mM Tris, pH 7.4; 125 mM NaCl; 0.2% Tween 20; 5% nonfat dry milk; and 0.1% sodium azide) for 30 min at room temperature, and then incubated for 2 h with anti-human COX-2, TIMP-1, N-cadherin, p-ERK1/2, p-TAK1, p-p38, p-Smad2 and GAPDH antibodies. After incubation with respective secondary antibodies and a final wash of the PVDF membranes, the immuno-reactive bands were developed by Amersham Enhanced Chemiluminescence (ECL) reagents and visualized/ photographed using an Image Reader (LAS-4000; Fujifilm, Japan).

PathScan p-Smad2 ELISA was also used to determine Smad2 phosphorylation. Briefly, 5 x 10^5^ SHED cells were seeded in 6-well plates overnight and then exposed to TGF-β1 (5 ng/ml) for 0-120 min. Medium was removed, cells were washed by phosphate-buffered saline (PBS) and cell lysate was isolated. Protein concentrations of cell lysates were measured by BioRad protein concentration assay kit. The equal amounts of proteins (600 μg) were utilized to study the p-Smad2 levels (activation) by the procedures of PathScan p-Smad2 ELISA [34].

### Cell number analysis

Briefly, 5 x 10^4^ SHED cells/well in 6-well plates were incubated in serum-free DMEM containing various concentrations of TGF-β1 (0, 0.1, 0.5, 1, 5, 10 ng/ml) for 5 days. The number of viable cells was estimated by MTT assay. In short, 0.5 mg/ml MTT was added into each well and cultured for additional 2 hours. Culture medium was aspirated, and the generated purple formazan was dissolved with DMSO. The dissolved formazan solution was forward to a 96-well plate and read against blank (DMSO) at OD540 with the Dynatech Microwell plate reader. Results were shown as percentage of control (as 100%). In some experiments, cells were pretreated with SB431542 (ALK5/Smad2 signaling inhibitor), 5z-7-oxozeaenol (TAK1 inhibitor), U0126 (MEK/ERK inhibitor), SB203580 (p38 inhibitor) for 30 min before the addition of TGF-β1 (5 ng/ml), and then co-incubated for 5 days before MTT assay.

### Collagen content analysis

Collagen content in cultured SHED was determined by Sircol^TM^ collagen assays kit as described before [34, 77]. In brief, 1 x 10^5^ SHED/well in 24-well cultured plates were incubated in serum-free DMEM containing TGF-β1 (0-10 ng/ml), with/without SB431542, 5z-7-oxozeaenol, U0126 or SB203580 for 5 days. Culture medium was removed and cells were washed with PBS. Then 50 μl of 0.5 M acetic acid was added for cell fixation and cells were finally stained with 200 μl of Sircol dye reagent for 30 min. The amount of sirius red binding to collagen was extracted by 0.2 ml alkaline reagent (0.5 M NaOH) for 10 min. A standard collagen solution was utilized for calibration of the standard curve. The optical density (OD) of samples was measured against blank at a wavelength of 540 nm by a Dynatech Microwell plate reader.

### Alkaline phosphatase (ALP) assay

### ALP staining

ALP staining was performed as described before [34]. Human SHED (1 x 10^5^ cells/well) in 24-well plates were incubated in different concentration of TGF-β1 (0-10 ng/ml) with/without SB431542, 5z-7oxozeneanol, SB203580 and U0126 for 5 days in DMEM containing 10% FBS. Culture medium was collected for ELISA. Then cells were fixed in 2% paraformaldehyde for 20 min and washed twice with PBS. Cells were eventually stained by a freshly-prepared stock substrate solution (10 mg fast blue 2’,5’-diethoxybenzanilide (BB) salt/50 ml ddH_2_O, 3.02 g Tris-base [pH 9.1] comprising 0.015 g naphthol AS phosphate and 250 μl of N,N dimethyl formamide) for 5-30 minutes in the dark condition. The results of cellular ALP staining were observed and photographed by a camera.

### Quantitative ALP activity analysis

In brief, 1 x 10^5^ SHED/well in 24-well cultured plates were exposed to DMEM with 10% FBS containing various concentration of TGF-β1 (0-10 ng/ml) for 5 days. Medium was collected and then 250 μl/well of extraction buffer (0.5% Triton X-100 + 2 mM MgCl_2_) was added for lysis of cells on ice for 30 min and supernatant was used for ALP activity assay. ALP activity was determined by measuring the amount of p-nitrophenol production (nM/min/well) using ALP activity assay kits (Sigma Chemical Company, St Louis, MO, USA) [34, 35].

### Statistical analysis

Three or more independent experiments were conducted. Statistical difference between control and experimental groups was analyzed by One-way ANOVA and post hoc Tukey test using the SPSS 10.0 software for Windows. A p value < 0.05 was regarded to show a statistically significant difference between groups.

### Ethics approval

This study is approved by Ethics Committee of National Taiwan University Hospital
